# Opinion: Advancing Embodiment Research From a Developmental Point of View

**DOI:** 10.3389/fnsys.2021.748335

**Published:** 2021-08-26

**Authors:** Robert Lickliter

**Affiliations:** Department of Psychology, Florida International University, Miami, FL, United States

**Keywords:** embodiment, developmental systems, constraints, phenotypic stability, phenotypic variability

## Introduction

Psychologists, neuroscientists, and philosophers are increasingly promoting the perspective that cognition is best characterized as something individuals do in practice, through their embodiment and embeddedness in the world. Contrary to the assumptions of the information processing paradigm dominant in the “cognitive revolution” of the last century, cognition is now less likely to be seen as the computational processing of representations in the brain. Rather, as (Engel et al., 2013, p. 202) have proposed, cognition is now more often seen as “skillful know-how in situated and embodied action.” This action-oriented paradigm is increasingly evident in contemporary psychological science (e.g., Overton et al., 2008; Witherington and Heying, 2013; Shapiro, 2014; Marshall, 2015; Crippin and Schulkin, 2020; Dess, 2021), and there is growing acknowledgment that having the *body in action* as a central focus for theories of perception and cognition will both advance and help unify psychological science (e.g., Thelen, 2000; Overton, 2008; Glenberg et al., 2013).

In their clear and persuasive article, Lux et al. (2021) have provided a significant contribution to organizing and integrating this conceptual turn, both within and beyond psychology. Their paper provides a well-reasoned developmental framework for bridging timescales and levels of analysis within and across the various disciplines concerned with *embodiment*. Lux et al. use of a developmental framework for this integrative effort is, to my way of thinking, essential for successfully advancing the impact and application of embodiment research within and beyond psychology. The process-relational paradigm that characterizes contemporary developmental science views individuals as active agents in constructing knowledge through their embodiment and embeddedness in the physical, biological, social, and cultural environments in which they develop (Overton, 2008). From this view cognitive skills, like all of development, results from the specific activities, experiences, conditions, and resources individuals encounter and take part in as they live their lives. This developmental framework emphasizes the fundamental relations among body, brain, and world and recognizes that perceptual, motor, emotional, and cognitive functioning are inherently co-dependent. This deeply situated perspective is a far cry from the computational paradigm of cognitive science that held that cognitive functions are wholly realized by information processing mechanisms inside the brain.

## Taking a Developmental Point of View

Lux et al. effectively outline both the importance and the benefits of a developmental framework to a wide range of topics and concerns in contemporary embodiment research, including identifying and providing a nuanced examination of the “transmission hubs” between the multiple levels of activity involved in human development—genetic, epigenetic, cellular, neural, sensory, motor, perceptual, behavioral, social, and cultural. A particular strength of the developmental framework promoted by Lux et al. is that it employs explanatory pluralism to integrate the various approaches currently used in programs of research on embodiment, particularly the *agency* approach and the *environmental* approach. As Lux et al. note, integrating these two research approaches within a developmental framework can provide a range of dividends, including connecting different timescales of embodiment, relating different systems levels to one another, and clarifying disciplinary boundaries. Integrating these approaches can also allow a deeper appreciation of the fact that organisms are both independent and interdependent, are both subject and object, and actively construct their own organization through their actions and exchanges with their environment. As (Overton, 2008, p. 9) succinctly put it, “all development is explained by the action of the individual,” where action and experience become synonymous.

In this brief opinion, I focus on two of the many developmental themes that Lux et al. address in their overview of current embodiment research and its potential future directions. The first of these is how to account for the stability and variability of developmental outcomes, a concern of both developmental and evolutionary theory. The second theme has to do with the importance of constraints and how they can contribute to a fuller understanding of the developmental dynamics of embodiment.

Regarding the first theme, as Lux et al. point out, accounting for change and stability from a developmental perspective requires rethinking *process*. From a developmental point of view, the process responsible for phenotypic stability and the process responsible for phenotypic variability are one and the same, namely, the very process of development itself (Lickliter and Harshaw, 2010). This view proposes that the stability of phenotypic outcomes across individuals is found not because of the transmission of genetic programs or the transfer of internal blueprints, but because a range of similar internal and external conditions, collectively conceptualized as developmental resources, are reliably available to developing individuals. Variability of phenotypic outcomes relies on these same developmental resources, but because the internal and external conditions of development are not always the same, phenotypes will also be characterized by at least some variability or change, within and across individuals. Lux et al.' example of the hormonal feed-back loops underlying the stress response established during early development nicely illustrates this dynamic perspective. They note that the set points of up and down regulation of an individual's stress response depend on a range of resources, including the current organization of the nervous system, the individual's metabolic conditions, the specifics of available stimulation, and the nature of the stress experiences encountered during this early period. Although these resulting set points can become relatively stabilized over development, conditions of trauma, hormonal imbalance, or chronic stress can all potentially lead to a change of these set points. In this light, organisms have developed a range of strategies to manage aspects of their own or their offspring's environment to guide and regulate these types of developmental process. This active management, often provided by parental care, allows a degree of dependability of developmental resources during early development, while also allowing for flexibility and adaptability to changing conditions (see Stotz, 2010). For example, rat pups that receive relatively high levels of maternal licking and grooming following birth typically show less physiological and behavioral response to stress throughout the life span than do pups that receive lower levels of maternal grooming during early development (Champagne et al., 2003). Importantly, these patterns can shift when pups are cross-fostered to mothers with different levels of maternal care. In this example, a pup's internal state and its sensitivities are dependent on something outside of it, illustrating the key insight that embodiment is always relational.

A second theme relevant to advancing our understanding of embodiment across the life-span is the notion of constraints. In the most general sense, constraints work by modifying the probability of the occurrence of events and actions. Constraints are relational and simultaneously open up as well as close off possible outcomes (Juarrero, 1999). For example, at all stages of development an individual's body is constrained in its capacities and possibilities of action. Not all movement is possible. Motor activity is both limited and facilitated by the design of muscles and tendons, their flexibility, their relations with other muscles and joints, and their prior history of use. As result, developing organisms, as subjects of their own activity, constrain the dynamics that give rise to and maintain their motor, perceptual, cognitive, and behavioral traits and characteristics. In other words, an organism contributes to its own developmental course by its specific sensitivities, abilities, biases, and previous history, creating its own “effective” environment by the scope and constraints of its own activity in the world. Lux et al.' concern for accounting for gain and loss of function across the lifespan fits nicely into this dynamic perspective.

## Discussion

Lux et al. have provided an important addition to the embodiment literature, detailing a framework and heuristic that can take this diverse field into a more integrative and interdisciplinary future. Their well-exampled conceptual analysis of embodiment research and its possibilities for future directions provides a useful road map for how to reduce conflict and enhance communication and collaboration between the various levels and different disciplines involved in embodiment research. As Lux et al. make clear, embodiment research is transforming multiple disciplines across the life sciences and the developmental framework they have proposed provides important conceptual tools to advance this transformation, as well as moving research questions and designs to more deeply and effectively unpack the complex nature of the relations among biological, psychological, and cultural systems involved in human development across the life span.

## Author Contributions

RL conceptualized and wrote this Opinion.

## Conflict of Interest

The author declares that the research was conducted in the absence of any commercial or financial relationships that could be construed as a potential conflict of interest.

## Publisher's Note

All claims expressed in this article are solely those of the authors and do not necessarily represent those of their affiliated organizations, or those of the publisher, the editors and the reviewers. Any product that may be evaluated in this article, or claim that may be made by its manufacturer, is not guaranteed or endorsed by the publisher.
